# Typification and Nomenclature Notes on Twenty-Nine Names in *Asparagus* (Asparagaceae)

**DOI:** 10.3390/plants12132537

**Published:** 2023-07-03

**Authors:** Zhiyong Zhang, Muhammad Idrees, Fang Wang, Nan Zhang, Wennian Chen, Mushtaq Ahmad, Shazia Sultana, Yongqing Jiao, Xu Zheng, Meng Li, Zakaria H. Prodhan, Muhammad Arfan

**Affiliations:** 1 College of Life Science, Neijiang Normal University, Neijiang 641000, China; 10000674@njtc.edu.cn (F.W.); 10000675@njtc.edu.cn (W.C.); mushtaqflora@hotmail.com (M.A.); shaziaflora@hotmail.com (S.S.); rajugenetics2003@gmail.com (Z.H.P.); 2 Department of Plant Science, Faculty of Biological Sciences, Quaid-i-Azam University, Islamabad 45320, Pakistan; 3 Pakistan Academy of Science, Islamabad 45320, Pakistan; 4 State Key Laboratory of Wheat and Maize Crop Science, Center for Crop Genome Engineering, Longzi Lake Campus, College of Agronomy, Henan Agriculture University, Zhengzhou 450046, China; jiaoyongqing@henau.edu.cn (Y.J.); zhengxu@henau.edu.cn (X.Z.); 5 Co-Innovation Center for Sustainable Forestry in Southern China, College of Biology and the Environment, Nanjing Forestry University, Nanjing 210037, China; 6 Department of Botany, University of Education Lahore, Vehari Campus, Vehari 61100, Pakistan; m.arfan@ue.edu.pk

**Keywords:** asparagaceae, lectotypification, nomen novum, nomenclature

## Abstract

Nomenclatural types for twenty-nine names belonging to the genus *Asparagus* are typified and discussed. The following names are lectotypified: *A. altiscandens* Engl. & Gilg, *A. altissimus* Munby, *A. baumii* Engl. & Gilg, *A. benguellensis* Baker, *A. burchellii* Baker, *A. curillus* Buch.-Ham. ex Roxb., *A. deflexus* Baker, *A. duchesnei* L.Linden, *A. equisetoides* Welw. ex Baker, *A. fasciculatus* Thunb., *A. griffithii*, Baker, *A. homblei* De Wild., *A. kaessneri* De Wild., *A. lecardii* De Wild., *A. longicladus* N.E.Br., *A. longiflorus* Franch., *A. monophyllus* Baker, *A. palaestinus* Baker, *A. pastorianus* Webb & Berthel., *A. persicus* Baker, *A. poissonii* H.Perrier, *A. psilurus* Welw. ex Baker, *A. ritschardii* De Wild., *A. sapinii* De Wild., *A. scandens* Thunb., *A. schumanianus* Schltr. ex H.Perrier, *A. stellatus* Baker, *A*. *subfalcatus* De Wild., and *A. undulatus* (L.f.) Thunb. (synonym of *Dracaena undulata* L.f.). A new name, *Asparagus neofasciculatus*, is proposed as a replacement name for *A. fasciculatus* Thunb., which is an illegitimate later homonym of *A. fasciculatus* R.Br. The original protologue of these names and the original materials are evaluated. Nomenclature remarks discussing the selection of type specimens are given for each name, and known isotypes or isolectotypes are also cited. This information could be utilized as a reference for future taxonomic and systematic studies on *Asparagus* around the world.

## 1. Introduction

The genus *Asparagus* L. [1] belongs to the family Asparagaceae Juss. [2] of order Asparagales Link [3,4], and comprises about 160–300 species [5,6,7,8,9]. The genus is widespread across the Old World continents [5,10], and has been suggested to originate from Africa, especially South Africa and adjacent regions [6], subsequently spreading throughout the Old World (Asia and Europe) through intensive speciation and dispersal [11,12]. The genus *Asparagus* is characterized by perennial herbs or subshrubs that are dioecious or hermaphroditic, with short rhizomes; the main stems are climbing, spreading or erect and are often branched, with cladodes (leaflike stems) in the axils of the main stems and branches; cladodes are borne in clusters, rarely solitary or fasciculate, and are flat, 3-angled, or subterete. The leaves are tiny, appressed to the stem, scalelike, and spurred at the base, with spurs frequently extending into spines. The inflorescences are an axillary cluster of flowers, rarely a single flower, and occasionally an umbel or a raceme. Flowers are unisexual or bisexual (2–12 mm diameter), white or whitish, with articulate pedicel, and subtended by membranous bracteoles; campanulate or subglobose perianth, segments free or occasionally connate at base; six stamens, filaments usually adnate to perianth segments; anthers dorsifixed. The ovary is three-loculed, with a few ovules per locule; the berry contains one to a few seeds [7]. The majority of the species in this genus are ecologically and economically important for ornamental purposes (*A. densiflorus*, *A. asparagoides*, *A. plumosus*, *A. virgatus*, and *A. setaceus*), as food (*A. albus*, *A. officinalis, A. acutifolius*, and *A. maritimus*), as well as for medicine (*A. adscendens*, *A. cochinchinensis*, *A. verticillatus*, and *A. racemosus*) [13,14,15,16,17].

The infrageneric classification of *Asparagus* has been controversial, since Willdenow [18] described the genus *Myrsiphyllum* Willd., which included all bisexual species, with axillary flowers and flattened cladodes. Since then, various authors have either recognized *Asparagus* as a single genus [19,20] or separated it into two or three genera [10,21,22,23,24], based on differing viewpoints on the significance of certain morphological features (such as spines, flowers, and cladodes). Baker [6] provided another approach, splitting the genus into three subgenera: *Asparagopsis* (Kunth) Baker (hermaphroditic species), (Eu)*asparagus* Baker (dioecious species), and *Myrsiphyllum* (Willd.) Baker (hermaphroditic species). *Asparagopsis* was an illegitimate homonym in Kunth’s application because Montagne [25] had used it for a genus of the Rhodophycaceae (*Asparagopsis delilei* Montagne, now *Asparagopsis taxiformis* (Delile) Trevisan). Obermeyer [23] published a replaced name *Protasparagus* Oberm., and this approach was also used in the Obermeyer and Immelman [26] treatment of the family for the southern African flora.

In the first comprehensive revision of a substantial part of the species, Jessop [27] challenged Baker’s classification by recognizing 40 southern African taxa in a single genus, *Asparagus*. He disputed the division of *Asparagus* and *Asparagopsis* (as proposed by Kunth [21], and Baker [6]) because none of the species included in the subgenus *Asparagus* was dioecious according to Kunth [21]. Instead, based on what he considered natural groupings, Jessop [27] classified *Asparagus* into eight groups, with *Myrsiphyllum* remaining as one of the sections. Obermeyer [24] advocated dividing *Asparagus* into three subgenera (according to Kunth): *Asparagus*, *Myrsiphyllum*, and *Protasparagus*. The species in the subgenus *Asparagus* are all dioecious (Eurasian); however, the species in the latter two subgenera are all hermaphrodites and can be distinguished by differences in the shape of their flowers and the numbers of ovules [10,23]. The most recent taxonomic classification of the genus in the region, found in the flora of southern Africa [26], maintains this circumscription.

Malcomber and Demissew [19] concluded that the only difference was in flower sexuality. Since *Protasparagus* and *Asparagus* share the trait of having free filaments, it is not possible to separate them at the generic level or even to maintain them as separate subgenera. *Asparagus* and *Protasparagus* belong to the same subgenus. On the basis of connivant filaments as opposed to free ones in the remainder of the Asparagaceae, *Myrsiphyllum* could not be a separate genus. However, this trait was enough to identify the taxon as a subgenus. As a result, they suggested that the Asparagaceae contain only one genus, *Asparagus,* with the subgenera *Myrsiphyllum* and *Asparagus*, the latter of which includes the species that were formerly classified as *Protasparagus*. Fellingham and Meyer [28] rejected the subdivision, at least for the southern African species, because of the morphological traits used to differentiate subgenus *Myrsiphyllum* becoming less distinct when the species were not covered by Malcomber and Demissew [19] treatment. As a result, the last publication on the subject recognized an undivided genus, *Asparagus*, without an infrageneric classification. Phylogenetic analysis using DNA sequences data [29,30,31] confirmed its monophyletic origin, with sexual dimorphism and polyploidy as the main force of evolution as well as dioecious species evolved from hermaphrodite species [32].

Typification is an important starting point for research on nomenclature and taxonomy. The majority of the names in *Asparagus* were previously typified by Jessop [27], Doronkin et al. [33], Ferrer-Gallego [34], Forman [35], Kay et al. [36], Noltie [37], Obermeyer [24], Obermeyer and Immelman [26], Press and Shrestha [38], and Valdés [39,40]. In the course of an ongoing revision of the genus *Asparagus*, we summarized all the accepted names in *Asparagus* listed in POWO [9] and discovered that several validly published names had not been typified yet. As a result, these names are typified here for nomenclatural stability. We reviewed the original protologues and the type specimens of 29 names: *A. altiscandens* Engl. & Gilg, *A. altissimus* Munby, *A. baumii* Engl. & Gilg, *A. benguellensis* Baker, *A. burchellii* Baker, *A. curillus* Buch.-Ham. ex Roxb., *A. deflexus* Baker, *A. duchesnei* L.Linden, *A. equisetoides* Welw. ex Baker, *A. fasciculatus* Thunb., *A. griffithii*, Baker, *A. homblei* De Wild., *A. kaessneri* De Wild., *A. lecardii* De Wild., *A. longicladus* N.E.Br., *A. longiflorus* Franch., *A. monophyllus* Baker, *A. palaestinus* Baker, *A. pastorianus* Webb & Berthel., *A. persicus* Baker, *A. poissonii* H.Perrier, *A. psilurus* Welw. ex Baker, *A. ritschardii* De Wild., *A. sapinii* De Wild., *A. scandens* Thunb., *A. schumanianus* Schltr. ex H.Perrier, *A. stellatus* Baker, *A*. *subfalcatus* De Wild., and *A. undulatus* (L.f.) Thunb. (synonym of *Dracaena undulata* L.f.), which are typified and discussed here.

## 2. Materials and Methods

This research is based on the examination of the original protologues of all the accepted names in the *Asparagus* genus, and the relevant literature, monographs and flora were checked in order to search for possible types and confirm the typification status of the names. The following herbaria were checked by the authors for putative type material: BM, B, BR, BOL, FL, G, K, LE, LISU, M, MPU, P, S, SRGH and UPS (acronyms according to Thiers [41]). The accepted names are listed alphabetically and are bolded. All name bibliographic citations were verified using the original literature, as well as IPNI [42], Tropicos [43], POWO [9], and WFO [44].

The original protologue was compared to the original herbarium material, and the most complete and informative herbarium specimen was chosen. The lectotypifications were made in accordance with the guidelines and rules of *the International Code of Nomenclature for Algae*, *Fungi*, and *Plants* (Arts. 9.12 and 9.17 [45]). Furthermore, it was discovered that no one designated any lectotype in accordance with the Shenzhen code Arts. 9.3, 9.4 of the ICN, and that for purposes of priority (Art. 9.19, 9.20, and 10.5), the designation of a type is only achieved if the type is definitely accepted as such by the typifying author, if the type element is clearly indicated by direct citation including the term “type” (typus) or an equivalent, and, on or after 1 January 2001, if the typification statement includes the phrase “designated here” (hic designatus) or an equivalent under Art. 7.11 of the ICN [45].

## 3. Results and Discussion

### Typification of Asparagus Names

(1) ***Asparagus altiscandens*** Engl. & Gilg, Kunene-Sambesi-Exped. [Warburg] 196. 1903. Protologue citation: Am linken Kubango-Ufer, oberhalb des Quatiri, 1100 m ü. M., auf Sandboden und weissem, festem Kalkmergel. (Nr. 402, Blühend im 12 November 1899). **Type (lectotype designated here)**: Angola, Am linken Kubango-Ufer, oberhalb des Quatiri, 1100 m ü. M., auf Sandboden und weissem, festem Kalkmergel, 12 November 1899, *Baum 402* (B 10 0166855!; isolectotypes: BR0000008767066!, K000255678!, M0104346!, S-G-7314!). Image available at https://herbarium.bgbm.org/object/B100166855 (accessed on 15 April 2023).

Remarks. Engler and Gilg [46] provided the following locality information: “Am linken Kubango-Ufer, oberhalb des Quatiri, 1100 m ü. M., auf Sandboden und weissem, festem Kalkmergel. (Nr. 402, Blühend im 12 November 1899)” when describing *A. altiscandens*. Since then, no author, even mistakenly, has designated a lectotype. Engler’s and Gilg’s specimens were kept at B (however, mostly destroyed), and duplicates at BM, G, H, K, LE, P and WRSL [47]. We traced five duplicate specimens of “*Baum 402*, 12 November 1899”, kept at B (B 10 0166855), BR (BR0000008767066), K (K000255678), M (M0104346), and S (S-G-7314). None of these, according to Arts. 9.6 and 40 Note 1 of the ICN [45], should be treated as the holotype; instead, they are syntypes, and a lectotype must be chosen (Art. 9.17 of the ICN [45]). Hence, we designate the specimen “*Baum 402*, 12 November 1899” at B (B 10 0166855) as the lectotype here, since it has flowers, leaves and a solitary fruit.

(2) ***Asparagus altissimus*** Munby, Bull. Soc. Bot. France 2: 287. 1855. Protologue citation: Près le pont de l’Oued-Krouft environs du Sig (*Durando*). **Type (lectotype designated here)**: Algeria, Près le pont de l’Oued-Krouft environs du Sig, October 1850, *Durando s.n.* (K00255632!; isolectotypes: MPU001383!, MPU009865!, MPU001384, MPU001385!, MPU017499!, P00573353!, P00573354!, P00573355!, P00573356!). Image available at http://specimens.kew.org/herbarium/K00255632 (accessed on 15 April 2023).

= *A. altissimus* var. *asperulus* Maire, Bull. Soc. Hist. Nat. Afrique N. 29: 453. 1938.
= *A. altissimus* var. *foeniculaceus* (Lowe) Maire, Bull. Soc. Hist. Nat. Afrique N. 1931.
= *A. foeniculaceus* Lowe, J. Proc. Linn. Soc., Bot. 5: 44. 1860.
= *A. declinatus* Schoub., lagttag. Vextrig. Marokko: 173. 1800, *nom. illeg.*

Remarks. Munby [48] cited one collection: “*Durando s.n.*” as the type (first step) but did not specify the herbarium where the specimen was stored. Munby’s original materials were deposited at K and YRK [49]. We did not trace the specimen “*Durando s.n.*” at YRK. However, ten duplicate specimens were traced, one at K (K000255632), four at P (P00573353, P00573354, P00573355, and P00573356), and five at MPU (MPU009865, MPU001385, MPU001383, MPU001384 and MPU017499). All of these collections should be considered syntypes in accordance with Arts. 9.6 and 40 Note 1 of the ICN [45]; therefore, one of them must be chosen as the lectotype (Art. 9.17 of the ICN). The blooming specimen “*Durando s.n.*” at K (K000255632) is designated here as the lectotype (second step), since it is in better condition than the other samples and has many branches, leaves and flowers.

(3) ***Asparagus baumii*** Engl. & Gilg, Kunene-Sambesi-Exped. [Warburg] 196. 1903. Protologue citation: Rechtes Ufer des Okachitanda, 1500 m ü. M., auf Sandboden. (Nr. 150, Blühend im 25 September 1899). **Type (lectotype designated here)**: Angola, Rechtes Ufer des Okachitanda, 1500 m ü. M., auf Sandboden, 25 September 1899, *Baum 150* (B 10 0166853!; isolectotypes: BR0000008764065!, K000255667!). Image available at https://herbarium.bgbm.org/object/B100166853 (accessed on 15 April 2023).

Remarks. In the protologue, Engler and Gilg [46] mentioned the following locality information: “Rechtes Ufer des Okachitanda, 1500 m ü. M., auf Sandboden. (Nr. 150, Blühend im 25 September 1899)”, as the type (first step), but without mentioning the herbarium where the type specimen was kept. In addition, no author, even mistakenly, has identified a lectotype. We located four original materials of “*Baum 150*, 25 September 1899”, which were deposited at B (B 10 0166853), BR (BR 0000008764065), K (K000255667), and M (M0104347). All of these collections should be considered syntypes (ICN Arts. 9.6 and 40 Note 1 [45]), and one of them must be chosen as the lectotype (Art. 9.17 of the ICN). The specimen at M (M0104347) collected from Angola: (Am Chitanda unterh. Gondkapje, 25 November 1899) cannot be selected as the lectotype because the original protologue mentioned the date “25 September 1899”. According to Stafleu and Cowan [47], Engler’s and Gilg’s original materials were deposited at B. The blooming specimen “*Baum 150*, 25 September 1899” in B (B 10 0166853) is designated here as the lectotype (second step). The chosen specimen is in better condition than the other samples and has many branches, leaves and flowers.

(4) ***Asparagus benguellensis*** Baker, Trans. Linn. Soc. London, Bot. 1(5): 253. 1878. Protologue citation: Mossamedes et Huilla, in dumetis sylvestribus ad Mumpullo. Floret Julio (July) 1859. **Type (lectotype designated here)**: Angola, Mossamedes: Février, in dumetis sylvestribus ad Mumpullo, 5 July 1859, *Welwitsch 3872* (LISU222051!; isolectotypes: BM000911577!, LISU222052!). Image available at https://plants.jstor.org/stable/10.5555/al.ap.specimen.lisu222051 (accessed on 15 April 2023).

Remarks. In the protologue, Baker [50] cited information on two localities: Mossamedes et Huilla, in dumetis sylvestribus ad Mumpullo, but did not indicate any collector name or number. The systematic description of the Liliaceae (now Asparagaceae) in the late Dr. Welwitsch’s Angolan Herbarium is the focus of Baker’s paper. Stearn [51] asserts that the original specimens, notes, and descriptions by F.M.J. Welwitsch (1806–1872) were kept at the Botanic Garden of Lisbon Herbarium (LISU), the second-best set and a copy of the written materials should be housed at the Natural History Museum (BM) in London, while the remaining sets were donated to other organizations [51,52]. We traced two original materials collected from Mossamedes: “*Welwitsch 3872*”, deposited at LISU (LISU222051 and LISU222052), and BM (BM000911577), as well as another original material collected from Huilla: “*Welwitsch 38723*”, kept at LISU (LISU222053). All of these collections should be regarded as syntypes in accordance with ICN Arts. 9.6 and 40 Note 1 [45]; therefore, one of them must be chosen as the lectotype (Art. 9.12 of the ICN). The herbarium specimen “*Welwitsch 3872*” at LISU (LISU222051) is designated as the lectotype here, among the known collections, since LISU222051 is in better condition than the other samples and has many branches, leaves and flowers.

(5) ***Asparagus burchellii*** Baker, J. Linn. Soc., Bot. 14: 618. 1875. ≡ *Protasparagus burchellii* (Baker) Oberm., Fl. S. Afr. 5(3): 27. 1992. Protologue citation: Cap. Bonae Spei in aridis, *Burchell 2962*, *Zeyher (Asparagus 10)*, *Cooper 1574*. **Type (lectotype designated here)**: South Africa, Cap. Bonae Spei in aridis, 16 May 1813, *Burchell 2962* (K000255681!; isolectotype: G00168274!). Image available at http://specimens.kew.org/herbarium/K000255681 (accessed on 15 April 2023).

Remarks. Baker [6] cited three collections (*Burchell 2962*, *Zeyher (Asparagus 10), Cooper 1574*)” in the protologue when he described *A. burchellii* but did not indicate the type specimen. Baker’s original materials were kept at K and WELT, according to Stafleu and Cowan [47]. We located six original materials, two of them “*Burchell 2962*” at G (G00168274) and K (K000255681), three of them “*Zeyher 10*” at NBG (NBG 0095496-0 and NBG0198297-0) and PRE (PRE0053008-0), and one of them “*Cooper 1574*” at BM (BM000911592). However, we did not trace any available collections at WELT. All of these collections should be regarded as syntypes (Art. 9.6 of the ICN [45]), and one of them needs to be chosen as the lectotype (Art. 9.12 of the ICN). After carefully reviewing all the available collections, we choose the blooming specimen “*Burchell 2962*” in K (K000255681) as the lectotype. The chosen specimen is in better condition than the other samples and has leaves, a few flowers and one fruit.

(6) ***Asparagus curillus*** Buch.-Ham. ex Roxb., Fl. Ind. 2: 152. 1832 [*A. curillus* Buch.-Ham. ex Roxb., Hort. Bengal. 24. 1814 *nom. inval.*]. ≡ *Asparagopsis curilla* (Buch.-Ham. ex Roxb.) Kunth, Enum. Pl. 5: 102. 1850. ≡ *Protasparagus curillus* (Buch.-Ham. ex Roxb.) Kamble, J. Econ. Taxon. Bot. 15: 708 (1991 Publ. 1992). **Type (lectotype designated here)**: Nepal, without locality and date, *Roxburgh s.n.* (BR0000006885595!: isolectotype: BR0000006884932!). Image available at https://www.botanicalcollections.be/specimen/BR0000006885595 (accessed on 10 April 2023).

= *A. nepalensis* Baker, *J. Linn. Soc., Bot.* 14: 622. 1875. Protologue citation: A native of Nepal.

Remarks. The name *A. curillus* was first published by Roxburgh [53] in a list of the plants at the Calcutta Botanic Garden, without any further explanation or description, but with the location and author information: “Napaul; Dr. F. Buchanan, 1801”. Roxburgh validly published the name in Flora Indica, or Descriptions of Indian plants [54], along with a description and the statement that the species was “a native of Nepal” but without any reference to any specimen that would serve as a type in the protologue. Forman [35] selected the specimen at BR as the lectotype (with Suppl. specimen in Herb. Wall.), which should be considered a first step. Later, Press and Shrestha [38] selected the specimen at LINN-SM 600.9 as the lectotype, which should be considered a nomenclaturally superfluous lectotypification because Forman [35] had already selected the Roxburgh original material at BR. Two duplicate specimens of “*Roxburgh s.n.*”, kept at BR (BR0000006885595 and BR0000006884932), were traced, from which a second-step lectotype must be chosen (according to Arts. 8.3 and 9.15 [45]). Among these, the specimen “*Roxburgh s.n.*” at BR (BR0000006885595) is in better condition than the other samples, and this blooming specimen is designated as the lectotype here, superseding the Press and Shrestha [38] selection as provided by Art. 9.19 of the ICN [45].

We did not trace any specimen that was reported by Roxburgh [53] with the locality and source “Napaul; Dr. F. Buchanan, 1801”. It is quite possible that no herbarium specimens were collected from this shrub (or any others in the garden) until after Roxburgh left India early in 1813 and later died in Edinburgh in February 1815.

(7) ***Asparagus deflexus*** Baker, Trans. Linn. Soc. London, Bot. 1(5): 254. 1878. Protologue citation: Pungo Andongo. Floret Oct. **Type (lectotype designated here)**: Angola, Pungo Andongo, *Welwitsch 3874* (LISU222061!; isolectotype: LISU222062!). Image available at https://plants.jstor.org/stable/10.5555/al.ap.specimen.lisu222061 (accessed on 15 April 2023).

Remarks. Baker [50] cited the following locality information: “Pungo Andongo. Floret Oct.” in the protologue but did not indicate any collector name or number. The Liliaceae (now Asparagaceae) in the late Dr. Welwitsch’s Angolan Herbarium were described in detail in Baker’s study. Stearn [51] asserts that the original specimens, notes, and descriptions by Friedrich Martin Josef Welwitsch (1806–1872) were kept at Lisbon Botanic Garden Herbarium (LISU), the second-best set and a copy of the written materials should be housed at the Natural History Museum (BM) in London, while the remaining sets were donated to other institutions [51]. We located four original materials collected by Welwitsch from Angola: “*F. Welwitsch 3874*” deposited at K (K000255669), BM (BM000911580), and LISU (LISU222061 and LISU222062). The herbarium specimens at K (K000255669) and BM (BM000911580) were labeled with the locality “Angola, Lutet”; hence, they could not be chosen as the lectotype because the original protologue indicated the locality “Pungo Andongo”. Herein, we designate one of the specimens at LISU (LISU222061) as the lectotype (Art. 9.17 of the ICN [45]). The chosen specimen has the same locality of “Pungo Andongo” and has many branches, leaves and flowers.

(8) ***Asparagus duchesnei*** L.Linden, Semaine Hort. 4: 471. 1900. Protologue citation: without any information. **Type (lectotype designated here)**: Democratic Republic of Congo, without date, *Duchesne s.n.* (BR0000013289577!; isolectotype: BR0000008764393!, BR0000008761361!, BR0000005183067!). Image available at https://www.botanicalcollections.be/specimen/BR0000008761361 (accessed on 16 April 2023). 

Remarks. In the protologue, Linden [55] described a new ornamental species *A. duchesnei* but did not indicate any type information, instead mentioning the plant photo exhibited in Paris. Two original specimens “*Duchesne s.n*.” were traced, deposited at BR (BR0000008764393 and BR0000008761361). It is quite possible that Linden collected the herbarium specimens from this shrub or any others in the garden. We also traced two illustrations representing *A. duchesnei*, deposited at BR (BR0000005183067 and BR0000013289577). None of these four specimens should be considered a holotype; rather, they should be regarded as syntypes, and a lectotype must be chosen in accordance with Arts. 8.3 and 9.17 of the ICN [45]. One of the original illustrations at BR (BR0000013289577) is designated here as the lectotype because it is in better condition than the other samples.

(9) ***Asparagus equisetoides*** Welw. ex Baker, Trans. Linn. Soc. London, Bot. 1(5): 253. 1878. Protologue citation: Pungo Andongo, in arenosis ad ripas fluminis Cuenza. **Type (lectotype designated here)**: Angola, Pungo Andongo, Cuenz, 1 February 1857, *Welwitsch 3846* (LISU222050!; isolectotypes: BM000911578!, K000255658!). Image available at https://plants.jstor.org/stable/10.5555/al.ap.specimen.lisu222050 (accessed on 15 April 2023).

Remarks. In the protologue, Baker [50] cited information on one locality, “Pungo Andongo, in arenosis ad ripas fluminis Cuenza”, as the type when he described *A. deflexus*. In Baker’s work, the Liliaceae (now Asparagaceae) in the late Dr. Welwitsch’s Angolan Herbarium were described in detail. The original specimens, notes, and descriptions by F.M.J. Welwitsch (1806–1872) were preserved at LISU Herbarium, in the Botanic Garden of Lisbon, while the second-best copy and a set of the written materials remain at the Natural History Museum (BM) in London, and the remaining sets passed to other herbaria [51,52]. We traced two collections from Pungo Andonogo: “*Welwitsch 3846*”, deposited at BM (BM000911578), K (K000255659) and LISU (LISU222050), and the specimen “*Welwitsch 3847*” kept at K (K000255658) and LISU (LISU222049). All of these collections should be considered syntypes (ICN Arts. 9.6 and 40 Note 1 [45]), and one of them needs to be chosen as the lectotype (Art. 9.12 of the ICN [45]). The specimen “*F. Welwitsch 3846*” in LISU (LISU222050) is designated as the lectotype here. The chosen specimen is in better condition than the other materials and has many branches and leaves.

(10) ***Asparagus neofasciculatus*** M. Idrees, ***nom. nov*.**

Replaced name: *A. fasciculatus* Thunb., *Fl. Cap. (Thunberg) Ed. 1a,* 2: 329. 1820 *nom. illeg*., non *A. fasciculatus* R.Br., *Prodr. Fl. Nov. Holland.* 281. 1810. ≡ *Myrsiphyllum fasciculatus* (Thunb.) Oberm., Bothalia 15: 87. 1984. ≡ *A. falcatus* var. *fasciculatus* (Thunb.) Kuntze, Revis. Gen. Pl. 3(3): 315. 1898. **Type (lectotype designated here)**: South Africa, Cape of Good Hope: without precise locality, *Masson s.n.* (UPS:BOT:V-008447!). Image available at https://databas.evolutionsmuseet.uu.se/botanik/browserecord.php?-action=browse&-recid=210118 (accessed on 17 April 2023).

= *A. consanguineus* (Kunth) Baker, J. Linn. Soc., Bot. 14: 615. 1875.
= *Asparagopsis consanguinea* Kunth, Enum. Pl. [Kunth] 5: 76. 1850.
= *A. asiaticus* var. *pauciflorus* Scott Elliot, J. Linn. Soc. Bot. 29: 60. 1891.
= *A. confusus* Scot Elliot ex H.Perrier, Notul. Syst. (Paris) 5: 25. 1935.

Remarks. The name *A. fasciculatus* R.Br. [56] was described in 1810 based on a single collection from Australia: “*R. Brown 5663*”, kept in BM (BM000990612), E (E0068250) and K (K000901281), and is currently treated as a synonym of *A. racemosus* Willd. [9,57]. Later, Thunberg [58] published *A. fasciculatus* from Cape. Recently, Germishuizen and Meyer [59] and POWO [9] listed the latter species as accepted and gave its native range as extending from Namibia to Free State, Madagascar. According to the ICN; Art. 53.1 [45], *A. fasciculatus* Thunb. is an illegitimate later homonym of *A. fasciculatus* R.Br. Therefore, a new name, *Asparagus neofasciculatus* M. Idrees, is proposed as a replacement name here. The specific epithet derives from the prefix *neo*, meaning new, and *fasciculatus*, the epithet first used by Thunberg [58].

In the protologue, Thunberg [58] described *A. fasciculatus*, but did not indicate any specimen type. Three original materials of *A. fasciculatus* Thunb. were traced, one of them “*Masson s.n*.” kept at UPS (UPS:BOT:V-008447), and two of them “*Thunberg s.n.*” kept at UPS (UPS:BOT:V-008448 and UPS:BOT:V-008449). According to Jessop [27], two of Thunberg’s specimens at UPS did not match Thunberg’s description since they had well-developed spines, while the specimen “*Masson s.n*.” kept at UPS (UPS:BOT:V-008447) had all of the characteristics and was chosen as the holotype. However, the Jessop [27] designation of “*Masson s.n*.” should be treated as a lectotype instead of a holotype because Thunberg did not cite any type information. Hence, following Rec. 9A.3 of the ICN [45], we here choose the specimen “*Masson s.n*.” kept at UPS (UPS:BOT:V-008447) as the lectotype.

(11) ***Asparagus griffithii*** Baker, J. Linn. Soc. Bot. 14: 604. 1875. Protologue citation: Afghanistan ad Topchee, *Griffith 5856.* **Type (lectotype designated here)**: Afghanistan, Topchee, 1852, *Griffith 5856* (K000901205!). Image available at http://specimens.kew.org/herbarium/K000901205 (accessed on 15 April 2023).

Remarks. Baker [6] listed one collection from Afghanistan (Topchee) “*Griffith 5856*” as the type (first step) but did not specify the herbarium where the specimen was deposited. Baker’s original specimens were kept at K and WELT, according to Stafleu and Cowan [47]. We did not trace the specimen “*Griffith 5856*”at WELT; however, three original materials of “*Griffith 5856*” kept at K (K000901204, K000901205 and K000901206) were traced. According to ICN Arts. 8.3 and 40 Note 1 [45], none of them may be considered the holotype, but rather syntypes. The specimens at K (K000901204 and K000901206) were labeled with the collector number “*1134*” in anonymous handwriting, while the specimen at K (K000901204) indicated the locality “Bamean (Bamyan)”. Thus, neither specimen can be chosen as the lectotype because the original protologue mentioned the locality and source “Topchee: *Griffith 5856*”. Hence, the specimen “*Griffith 5856*” at K (K000901205) is designated here as the lectotype (ICN Art. 9.12). The chosen specimen has the same locality and source information, “Topchee: *Griffith 5856*”, and has leaves, flowers and fruits.

(12) ***Asparagus homblei*** De Wild., Repert. Spec. Nov. Regni Veg. 12: 292. 1913. Protologue citation: Ober-Katanga: 21 Février (February) 1912 (*Homblé, no. 172*—In termitière). **Type (lectotype designated here)**: République démocratique du Congo, Ober-Katanga: 21 Février (February) 1912, *Homblé 172* (BR0000008761545!; isolectotype: BR0000008760975!). Image available at https://www.botanicalcollections.be/specimen/BR0000008761545 (accessed on 17 April 2023).

Remarks. In the protologue, De Wildeman [60] mentioned one collection, “*Homblé 172”*, as the type (first step), but did not specify the herbarium where the specimen was stored. According to Stafleu and Cowan [47], De Wildeman’s original materials were kept at BR. We located two “*Homblé 172*” specimens at BR (BR0000008761545 and BR0000008760975). According to ICN Arts. 8.3 and 9.17 [45], both of these specimens are syntypes, and one of them must be chosen as the lectotype. The specimen of “*Homblé 172*” at BR (BR0000008761545) is designated here as the lectotype (second step). The chosen specimen has the same date and locality information and has many branches, leaves and fruits.

(13) ***Asparagus kaessneri*** De Wild., Repert. Spec. Nov. Regni Veg. 12: 293. 1913. Protologue citation: Zentral-Afrika-Seengebiet: Vallée de la Rusisi, 22 juillet (July) 1908 (*Kassner*, no. *3178*). **Type (lectotype designated here)**: Central Africa, Seengebiet: Vallée de la Rusisi, 22 Juillet (July) 1908, *Kassner 3178* (BR0000008760944!, isolectotype: BR0000008761064!). Image available at https://www.botanicalcollections.be/specimen/BR0000008760944 (accessed on 17 April 2023).

Remarks. De Wildeman [60] cited one collection in the protologue, “*Kassner 3178*”, as the type (first step), but without mentioning where the type was preserved. According to Stafleu and Cowan [47], De Wildeman’s original specimens were kept at BR. We located two duplicate materials “*Kassner 3178*” kept at BR (BR0000008760944 and BR0000008761064). According to ICN Arts. 8.3 and 9.17 [45], none of them should be regarded as the holotype, but they are syntypes, and a lectotype must be chosen (second step). One of the specimens at BR (BR0000008760944) is designated here as the lectotype. The chosen specimen is in better condition than the other samples and has many branches, leaves and flowers.

(14) ***Asparagus lecardii*** De Wild., Ann. Mus. Congo Belge, Bot. sér. 5, 1(1): 17. 1903. Protologue citation: Sénégal (*Lécard*, n. *31* et *36*). **Type (lectotype designated here)**: Sénégal, without date, *Lécard 31* (BR0000008765109!; isolectotypes: BR0000008761125!, BR0000008767363!, BR0000008761095!). Image available at https://www.botanicalcollections.be/specimen/BR0000008765109 (accessed on 17 April 2023).

Remarks. De Wildeman [61] cited two collections from Sénégal (*Lécard 31* et *36*) but did not indicate the specimen that might serve as the holotype. According to Stafleu and Cowan [47], De Wildeman’s original type materials were kept at BR. We did not trace the specimen “*Lécard 36*” at BR or any available herbarium. However, four original materials of “*Lécard 31*” kept at BR (BR0000008761125, BR0000008767363, BR0000008761095 and BR0000008765109) were traced. According to Arts. 8.3 and 9.12 of the ICN [45], all of these collections should be considered syntypes, and one of them needs to be chosen as the lectotype. Therefore, we here choose the specimen “*Lécard 31*” at BR (BR0000008765109) as the lectotype. The chosen specimen is in better condition than the other samples and has many branches, stems and flowers.

(15) ***Asparagus longicladus*** N.E.Br., Bull. Misc. Inform. Kew 1921(8): 298. 1921. ≡ *Protasparagus longicladus* (N.E.Br.) B.Mathew, Kew Bull. 44: 181. 1989. Protologue citation: Tropical Africa: Southern Rhodesia, Victoria Falls, 900 m, *F.A. Rogers 5523*. **Type (lectotype designated here)**: Africa (Zimbabwe): Southern Rhodesia, Victoria Falls, 900 m, 12 February 1912, *F.A. Rogers 5523* (K000255676!; isolectotypes: BOL140482!, K000255677!). Image available at http://specimens.kew.org/herbarium/K000255676 (accessed on 15 April 2023).

Remarks. Brown [62] cited the following locality and source information in the protologue as the type (first step): “Tropical Africa: Southern Rhodesia, Victoria Falls, 900 m, *F.A. Rogers 5523*”. Nicolas Edward Brown’s original materials were conserved at K [63]. We traced three duplicate specimens, one of them at BOL (BOL140482) and two of them kept at K (K000255676 and K000255677. Hence, these are all syntypes (Art. 8.3 of the ICN), and it is necessary to choose one of them as the lectotype (second step) (Art. 9.17 [45]). Here, one of the specimens at K (K000255676) is chosen as the lectotype. The chosen specimen is in better condition than the other samples and has many branches, leaves and flowers.

(16) ***Asparagus longiflorus*** Franch., Nouv. Arch. Mus. Hist. Nat., sér. 2, 7: 110. 1884. Protologue citation: Mongolie: Géhol, dans les lieux secs des montagnes; (no. *1766*). Fl. mai (May); fr. juin (June) 1864. **Type (lectotype designated here)**: China, Mongolie: Géhol, dans les lieux secs des montagnes, 1 May 1864, *A. David 1766* (P00687024!, isolectotype: P00687023!). Image available at http://coldb.mnhn.fr/catalognumber/mnhn/p/p00687024 (accessed on 15 April 2023).

Remarks. *A. longiflorus* was described by Franchet [64], who cited one collection, “*David 1776*, fl. Mai; fr. Juin 1864.”, as the type, without specifying the herbarium where the type was preserved. According to Stafleu and Cowan [47], Franchet worked at P. We located two collections: one of them is the flowering specimen “*David* 1776, fl. May 1864” kept at P (P00687024), and another is the fruiting specimen “*David* 1776, fr. June 1864” kept at P (P00687023). Both collections should be regarded as syntypes (Art. 9.6 of the ICN [45]), and one of them needs to be chosen as the lectotype (Art. 9.12 of the ICN). Therefore, we choose the flowering specimen of “*David* 1776, fl. May 1864” at P (P00687024) as the lectotype. The chosen specimen has the same locality of “Mongolie: Géhol” and has many branches, leaves and flowers.

(17) ***Asparagus monophyllus*** Baker, J. Linn. Soc., Bot. 14: 604 1875. Protologue citation: Beloochistan superior, 1851, *Stocks 1114* ex parte. **Type (lectotype designated here)**: Pakistan, Beloochistan (Balochistan), 1851, *Stocks 1114* (K000901201!, isolectotypes: K000901202!, K000901203!). Image available at http://specimens.kew.org/herbarium/K000901201 (accessed on 15 April 2023).

Remarks. In the protologue of *A. monophyllus*, Baker [6] cited one collection, “*Stocks 1114* ex parte”, without specifying the herbarium where the type was preserved. It is commonly known that Baker’s original specimens were kept at K and WELT [47]. Three materials were traced at K (K000901201, K000901202 and K000901203); however, we did not trace the specimen “*Stocks 1114*” at WELT. According to Art. 40 Note 1 of the ICN [45], all of these are syntypes. Hence, a lectotype must be chosen (Art. 9.17 of the ICN). Thus, one of the specimens kept at K (K000901201) is designated here as the lectotype. The chosen specimen is in better condition and has many branches, leaves and fruits.

(18) ***Asparagus palaestinus*** Baker, J. Linn. Soc., Bot. 14: 602. 1875. Protologue citation: Palaestina ad Huleh et ad vada fluminis Jordan, *Hayne*. **Type (lectotype designated here)**: Palaestina (Palestine), Huleh, April 1872, *Hayne s.n.* (K000901189!). Image available at http://specimens.kew.org/herbarium/K000901189 (accessed on 15 April 2023).

= *A. lownei* Baker, J. Linn. Soc., Bot. 14: 601. 1875.

Remarks. In the protologue, Baker [6] cited information on two different localities, “Palaestina ad Huleh et ad vada fluminis Jordan, *Hayne*” but did not indicate any specimen type. Since then, no author, even mistakenly, has selected a lectotype. Baker’s original specimens were kept at K and WELT, according to Stafleu and Cowan [47]. We located two original specimens, one of them from the locality “Huleh: *Hayne s.n.*” at K (K000901189), and another from the locality “Jordan: *Hayne s.n*.” at K (K000901190); however, we did not find the original materials among any of the available collections at WELT. Both collections should be regarded as syntypes (Art. 9.6 of the ICN [45]), and one of them needs to be chosen as the lectotype (Art. 9.12 of the ICN). Among these two collections, the original material “Huleh, *Hayne s.n*.” kept at K (K000901189) is selected as the lectotype. The chosen specimen is in better condition than the other materials and has many branches, leaves and flowers.

(19) ***Asparagus pastorianus*** Webb & Berthel., Hist. Nat. Iles Canaries (Phytogr.). iii: 329. t. 229. 1847. ≡ *Asparagopsis alba* var. *pastoriana* (Webb & Berthel.) Ball, Spic. Moroc.: 696. 1878. Protologue citation: in exsicc. *Bourgeau n. 210.* **Type (lectotype designated here)**: Spain, in exsicc. 1845, *Bourgeau 210* (FI011964!; isolectotype: Fl011965!). Image available at https://plants.jstor.org/stable/10.5555/al.ap.specimen.fi011964 (accessed on 15 April 2023).

Remarks. *A. pastorianus* was described by Webb and Berthelot [65], who cited one collection from Spain, “*Bourgeau 210*”, as the type, without specifying the herbarium where the type was preserved. In addition, no author, even mistakenly, has selected a lectotype. According to Stafleu and Cowan [66], Webb’s main materials were deposited at FI, and Berthelot’s original materials were deposited at B, FI, L, LY and P. We located two duplicate specimens of “*Bourgeau 210*”, kept at FI (FI011964 and FI011965). According to ICN Arts. 8.3 and 40 Note 1 [45], these are syntypes, and the name *A. pastorianus* needs lectotypification (Art. 9.17 of the ICN). Hence, we designate the specimen “*Bourgeau 210*” at FI (FI011964) as the lectotype, since it is in better condition than the other materials and has many branches, leaves and flowers.

(20) ***Asparagus persicus*** Baker, J. Linn. Soc., Bot. 14: 603. 1875. Protologue citation: Persia borealis ad radices montis Demavend prope pagum Ask, *Kotschy 365*. **Type (lectotype designated here**): Iran, ad radices montis Demawend (Mount Damavand) prope pagum Ask, 23 June 1843, *Kotschy 365* (K000901216!; isolectotypes: G00165455!, G00165456!, K000901217!, LE00010990!, LE00010991!). Image available at http://specimens.kew.org/herbarium/K000901216 (accessed on 15 April 2023).

= *A. leptophyllus* Schischk., Izv. Tomsk. Gosud. Univ. 81: 434. 1928.
= *A. oligophyllus* Baker, J. Linn. Soc., Bot. 14: 604. 1875.

Remarks. Baker [6] cited one collection, “*Kotschy 365*”, in the protologue, but did not indicate the herbarium where the specimen was stored. Since then, no author, even mistakenly, has designated a lectotype. According to Stafleu and Cowan [47], Baker’s main specimens were kept at K and WELT. We traced five duplicate specimens of “*Kotschy 365*”, two of them deposited at G (G00165455 and G00165456), two at K (K000901216 and K000901217) mounted on a single sheet, and two at LE (LE00010990 and LE00010991); however, we did not trace the specimen “*Kotschy 365*” at WELT. All of these duplicate specimens should be considered syntypes, and one of them needs to be chosen as the lectotype (Article 9.12 of the ICN) [45]. The specimen “*Kotschy 365*” at K (K000901216) is designated here as the lectotype, since it is in better condition than the other samples and has leaves and flowers, while the other specimen at K (K000901217) was labeled with anonymous handwritten notes that the specimen with berries seemed different, although Baker considered it the same as (1), from which it differs notably in its ascending branches and more numerous and slender cladodes.

(21) ***Asparagus poissonii*** H.Perrier, Notul. Syst. (Paris) 5: 21. 1935. Protologue citation: Domaine du S.W.: env. de Tuléar (*Poisson*. n. 4, 1 décembre 1916). **Type (lectotype designated here)**: Madagascar, environs de Tuléar, 1 décembre (December) 1916, *Poisson 41* (P00328170!; isolectotype: P00328171!). Image available at http://coldb.mnhn.fr/catalognumber/mnhn/p/p00328170 (accessed on 16 April 2023).

Remarks. Perrier [67] cited one collection from Madagascar: “*Poisson 4*” as the type but did not indicate the herbarium that housed the original specimen. No author, even mistakenly, has chosen a lectotype. Perrier’s original materials were deposited at P [68]. We did not find any potential original material numbered as *Poisson 4* among the available collections at P. However, two original specimens “*Poisson 41*”, kept at P (P00328170 and P00328171), were traced with the following description: “Coll. H. Poisson, Madagascar. Type”. The location and date matched the original protologue and were annotated with anonymous handwriting as *A. possonii* sp. n. “Type”. According to ICN Arts. 8.3 and 40 Note 1 [45], none of these specimens can be considered the holotype, but are rather syntypes, and one of them must be chosen as the lectotype (Art. 9.17 of the ICN) [45]. We designate one of the blooming specimens at P (P00328170) as the lectotype here. The chosen specimen is in better condition than the other samples and has many branches, leaves and flowers.

(22) ***Asparagus psilurus*** Welw. ex Baker, Trans. Linn. Soc. London, Bot. 1(5): 253. 1878. Protologue citation: Pungo Andongo, in dumetis Cuazensibus, frequens. Floret Maio (1 May 1857). **Type (lectotype designated here)**: Angola, Pungo Andongo, 1 May 1857, *Welwitsch 3870* (LISU222055!; isolectotypes: BM000911576!, K000255653!, LISU222056!). Image available at https://plants.jstor.org/stable/10.5555/al.ap.specimen.lisu222055 (accessed on 15 April 2023).

= *A. bechuanicus* Baker, D.Oliver & auct. suc. (eds.), Fl. Trop. Afr. 7: 429. 1898.
= *Protasparagus bechuanicus* (Baker) Oberm., Fl. S. Afr. 5(3): 33. 1992.

Remarks. Baker [50] cited one collection, “Pungo Andongo, in dumetis Cuazensibus, frequens” in the protologue when he described *A. psilurus*. Stearn [51] asserts that the original specimens of F.M.J. Welwitsch (1806–1872) were kept at LISU Herbarium (Lisbon Botanic Garden), while the second-best set and a copy of the written material were kept at BM in London, and the remaining sets or a copy were donated to other organizations [51]. We located four collections from the same locality, collected by Welwitsch from Pungo Andongo: one collection “*F. Welwitsch 3868*” deposited at K (K000255657) and LISU (LISU222057), the second collection “*F. Welwitsch 3869*” at K (K000255656) and LISU (LISU222058), the third collection “*F. Welwitsch 3870*” at BM (BM000911576), K (K000255653) and LISU (LISU222055 and LISU222056), and the fourth collection “*F. Welwitsch 3871*” at K (K000255654) and LISU (LISU222054). None of them should be considered the holotype, but they are all syntypes (Arts. 9.6 and 40 Note 1 of the ICN) [45]. Among all the available collections, one of them must be chosen to serve as the lectotype (Art. 9.12 of the ICN) [45]. Hence, we choose one of the specimens “*F. Welwitsch 3870*” in LISU (LISU222055) as the lectotype. The chosen specimen is in better condition than the other samples and has many branches, leaves and flowers.

(23) ***Asparagus ritschardii*** De Wild., Contr. Fl. Katanga, Suppl. 3: 98. 1930. Protologue citation: Aux environs de l’Arboretum extensions, sur termitière, vers 1300 m. d’altitude, 3 octobre 1927 (*F. Ritschard, n. 1501*—Nom ind.: Kakoba Makanga [Kib.]). **Type (lectotype designated here)**: République démocratique du Congo, Aux environs de l’Arboretum extensions, sur termitière, 3 octobre 1927, *F. Ritschard 1501* (BR0000008761705!, isolectotype: BR0000008761736!). Image available at https://www.botanicalcollections.be/specimen/BR0000008761705 (accessed on 17 April 2023).

Remarks. De Wildeman [69] cited one collection in the protologue, “*F. Ritschard 1501*”, as the type (first step); however, he did not specify where the original material was preserved. De Wildeman’s main materials were kept at BR (according to Stafleu and Cowan [47]). Two original materials were traced at BR (BR0000008761705 and BR0000008761736). Therefore, one of the specimens at BR (BR0000008761705) is designated as the lectotype (second step), according to Arts. 8.3 and 9.17 of the ICN [45]. The chosen specimen is in better condition than the other samples and has many branches, stems, leaves and flowers.

(24) ***Asparagus sapinii*** De Wild., Compagnie du Kasai 267. 1910. Protologue citation: Bienge dans la plaine, octobre 1907 (*A. Sapin*). **Type (lectotype designated here)**: République démocratique du Congo, Bienge dans la plaine, octobre (October) 1907, *A. Sapin C42* (BR0000008766038!; isolectotype: BR0000008761767!). Image available at https://www.botanicalcollections.be/specimen/BR0000008766038 (accessed on 17 April 2023).

Remarks. De Wildeman [70] cited the following information in the protologue: “Bienge dans la plaine, octobre 1907 (*A. Sapin*)”, without specifying the herbarium where the specimen was stored. In addition, no author, even mistakenly, has chosen a lectotype. De Wildeman’s main specimens were kept at BR [47]. We located three duplicate specimens, one of them, “*A. Sapin s.n*.”, kept at MPU (MPU017500), and two of them, “*A. Sapin C42*”, kept at BR (BR0000008766038 and BR0000008761767). None of them can be considered a holotype in accordance with Arts. 9.6 and 40 Note 1 (ICN) [45], but they are all syntypes; hence, one of them needs to be chosen as the lectotype (Art. 9.17 of the ICN) [45]. Herein, the blooming specimen “*A. Sapin C42*” at BR (BR0000008766038) is designated as the lectotype, since it is in better condition than the other materials and has many branches, leaves and flowers.

(25) ***Asparagus scandens*** Thunb., Prodr. Pl. Cap. 1: 66. 1794. ≡ *Asparagopsis scandens* (Thunb.) Kunth, Enum. Pl. 5: 78. 1850. ≡ *Myrsiphyllum scandens* (Thunb.) Oberm., Bothalia 15: 86. 1984. Protologue citation: without any type information. **Type (lectotype designated here)**: South Africa, Cape of Good Hope, without precise locality, *Thunberg s.n.* (UPS:BOT:V-008461!; isolectotype: UPS:BOT:V-008462!). Image available at https://databas.evolutionsmuseet.uu.se/botanik/browserecord.php?-action=browse&-recid=210132 (accessed on 17 April 2023).

= *A. pectinatus* Redouté, Liliac. 7: t. 407. 1813.

Remarks. Thunberg [71] has described the name *A. scandens* but did not indicate any type information. Jessop [27] mentioned the specimens “*Thunberg s.n*.” at UPSa, b, BOL, and PRE as syntypes. We did not locate any specimen of “*Thunberg s.n*.” at the BOL or PRE herbaria. However, we located two original materials deposited at UPS (UPS:BOT:V-008461 and UPS:BOT:V-008462); thus, according to the ICN (Art. 8.3 [45]), they are syntypes and one of them needs to be chosen as the lectotype (ICN Art. 9.17) [45]. Hence, we choose the specimen “*Thunberg s.n.*” in UPS (UPS:BOT:V-008461) as the lectotype here, since it is in better condition than the other samples.

(26) ***Asparagus schumanianus*** Schltr. ex H.Perrier, Notul. Syst. (Paris) 5: 23. 1935. Protologue citation: Plantae Schlechterianae Austro-Africanae, Iter Secundum, no. 10364. Regio occidentalis, Zwarteberg, 7-IV-1897. **Type (lectotype designated here)**: South Africa, Plantae Schlechterianae Austro-Africanae. Regio occidentalis, Zwarteberg, 7 April 1897, *Schlechter 10364* (LE00010997!, isolectotype: S-G-7313!). Image available at http://re.herbariumle.ru/00010997 (accessed on 16 April 2023).

Remarks. Perrier [67] cited one collection: “*Schlechter 10364*” as the type in the protologue but did not specify the herbarium where the type specimen was housed. In addition, no author, even mistakenly, has chosen a lectotype. We traced two duplicate specimens, “*Schlechter 10364*”, kept at LE (LE00010997) and S (S-G-7313); thus, they are syntypes and one of them must be chosen as the lectotype (ICN; Art. 9.12) [45]. Herein, the specimen “*Schlechter 10364*” at LE (LE00010997) is designated as the lectotype since it is in better condition and has many branches, leaves, inflorescences and flowers.

(27)***Asparagus stellatus*** Baker, J. Linn. Soc., Bot. 14: 612. 1875. Protologue citation: C. B. Spei, *Drège 8589*, *Cooper 622*. **Type (lectotype designated here)**: South Africa, C. B. Spei, 1861, *Cooper 622* (K000255713!; isolectotypes: BM000911597!, K000255712!). Image available at http://specimens.kew.org/herbarium/K000255713 (accessed on 15 April 2023).

Remarks. Baker [6] cited two collections, “*Drège 8589* and *Cooper 622*” in the protologue, but did not indicate the specimen that might serve as the holotype. Jessop [27] designated the two collections of “Albert Div.”, *Cooper 622* (K) and Aliwal North, Witteberge *Drège 8589* (K) as syntypes. According to Stafleu and Cowan [47], Baker’s original materials were kept at K and WELT. We located seven original materials: one of them, “*Drege 8589*”, kept at G (G00168282), K (K000255709), L (L0041373) and S (S06-4761), and the specimens “*Cooper 622*” kept at BM (BM000911597) and K (K000255712 and K000255713); however, we did not trace the specimens “*Drège 8589* or *Cooper 622*” at WELT. All of these collections should be regarded as syntypes (Art. 9.6 of the ICN [45]), and one of them needs to be chosen as the lectotype (Art. 9.12 of the ICN). Hence, we designate the blooming specimen “*Cooper 622*” at K (K000255713) as the lectotype. The chosen specimen is in better condition than the other samples and has many branches, leaves and flowers.

(28) ***Asparagus subfalcatus*** De Wild., Pl. Bequaert. 1: 42. 1921. Protologue citation: Kabare, 19 août 1914, *J. Bequaert,* n. *5365.* Steppe herbeuse des bords du lac. **Type (lectotype designated here**): République démocratique du Congo, Kabare, steppe herbeuse des bords du lac, 19 août 1914, *J. Bequaert 5365* (BR0000008761422!; isolectotype: BR0000008761453!). Image available at https://www.botanicalcollections.be/specimen/BR0000008761422 (accessed on 17 April 2023).

Remarks. De Wildeman [72] cited one collection in the protologue, “*J. Bequaert 5365*”, without mentioning the herbarium where the type specimen was stored. Tropicos [43] lists “*J. Bequaert 5365*” as the type at BR (first step). According to Stafleu and Cowan [47], De Wildeman’s main original materials were kept at BR. Two duplicate specimens kept at BR (BR0000008761422 and BR0000008761453) were traced. Both of these specimens should be considered syntypes (Art. 8.3 of the ICN) [45], and the name *A. subfalcatus* needs lectotypification (the ICN Art. 9.17) [45]. Therefore, one of the specimens at BR (BR0000008761422) is designated here as the lectotype (second step), since it is in better condition and has many branches, leaves and flowers.

(29) ***Asparagus undulatus*** (L.f.) Thunb., Prodr. Pl. Cap. 1: 66. 1794. ≡ *Dracaena undulata* L.f., Suppl. Pl. 203. 1782. ≡ *Myrsiphyllum undulatum* (L.f.) Schltdl. ex Kunth, Enum. Pl. 5: 109. 1850. Protologue citation: “Habitat in Cap. bonae spei. *Thunberg*”. **Type (lectotype designated here)**: South Africa, Cape of Good Hope, without precise locality, *Thunberg s.n.* (UPS:BOT:V-008465!; isolectotype: UPS:BOT:V-008466!). Image available at https://databas.evolutionsmuseet.uu.se/botanik/browserecord.php?-action=browse&-recid=210136 (accessed on 17 April 2023).

Remarks. Linnaeus f. [73] described D. undulata in 1782 and cited the following information: “Habitat in Cap. bonae spei. Thunberg”. Later, Thunberg [71] proposed a new combination, “A. undulata”, when transferring the name D. undulata to Asparagus and cited the basonym reference as D. undulata. Jessop [27] selected the specimen “Thunberg s.n.” kept at UPS as the holotype (first step). We traced two duplicate specimens kept at UPS (UPS:BOT:V-008465 and UPS:BOT:V-008466). According to the ICN (Art. 8.3 [45], they are syntypes, and one of them needs to be chosen as the lectotype (Art. 9.17 of the ICN) [45]. Herein, we choose the specimen “Thunberg s.n.” in UPS (UPS:BOT:V-008465) as the lectotype of the name of Dracaena undulata (second step), since it is in better condition than the other samples.

## 4. Conclusions

In this study, all names in the genus *Asparagus* were reviewed for proper typification and nomenclatural clarification. This research highlighted the examination of the original protologues and type specimens of some names in *Asparagus* that lack typification in order to assist with a new circumscription of this genus and to contribute to the stability of biological nomenclature. We revisited 29 lectotypifications and proposed a new name, *Asparagus neofasciculatus*, as a replacement name for the illegitimate name *A. fasciculatus* Thunb., a later homonym of *A. fasciculatus* R.Br. This study will be helpful for future research on taxonomy, nomenclature, and the systematic study of the genus *Asparagus* globally.

## Data Availability

All data are available within the article.
